# Radiomics Features Predict *Telomerase Reverse Transcriptase* Promoter Mutations in World Health Organization Grade II Gliomas *via* a Machine-Learning Approach

**DOI:** 10.3389/fonc.2020.606741

**Published:** 2021-02-11

**Authors:** Shengyu Fang, Ziwen Fan, Zhiyan Sun, Yiming Li, Xing Liu, Yuchao Liang, Yukun Liu, Chunyao Zhou, Qiang Zhu, Hong Zhang, Tianshi Li, Shaowu Li, Tao Jiang, Yinyan Wang, Lei Wang

**Affiliations:** ^1^ Beijing Neurosurgical Institute, Capital Medical University, Beijing, China; ^2^ Department of Neurosurgery, Beijing Tiantan Hospital, Capital Medical University, Beijing, China; ^3^ Department of Pathology, Beijing Tiantan Hospital, Capital Medical University, Beijing, China; ^4^ Department of Neuroradiology, Beijing Tiantan Hospital, Capital Medical University, Beijing, China

**Keywords:** low-grade glioma, machine-learning, nested cross-validation, radiomics, *TERT* promoter mutation

## Abstract

The detection of mutations in telomerase reverse transcriptase promoter (p*TERT*) is important since preoperative diagnosis of p*TERT* status helps with evaluating prognosis and determining the surgical strategy. Here, we aimed to establish a radiomics-based machine-learning algorithm and evaluated its performance with regard to the prediction of mutations in p*TERT* in patients with World Health Organization (WHO) grade II gliomas. In total, 164 patients with WHO grade II gliomas were enrolled in this retrospective study. We extracted a total of 1,293 radiomics features from multi-parametric magnetic resonance imaging scans. Elastic net (used for feature selection) and support vector machine with linear kernel were applied in nested 10-fold cross-validation loops. The predictive model was evaluated by receiver operating characteristic and precision-recall analyses. We performed an unpaired t-test to compare the posterior predictive probabilities among patients with differing p*TERT* statuses. We selected 12 valuable radiomics features using nested 10-fold cross-validation loops. The area under the curve (AUC) was 0.8446 (95% confidence interval [CI], 0.7735–0.9065) with an optimal summed value of sensitivity of 0.9355 (95% CI, 0.8802–0.9788) and specificity of 0.6197 (95% CI, 0.5071–0.7371). The overall accuracy was 0.7988 (95% CI, 0.7378–0.8598). The F1-score was 0.8406 (95% CI, 0.7684–0.902) with an optimal precision of 0.7632 (95% CI, 0.6818–0.8364) and recall of 0.9355 (95% CI, 0.8802–0.9788). Posterior probabilities of p*TERT* mutations were significantly different between patients with wild-type and mutant *TERT* promoters. Our findings suggest that a radiomics analysis with a machine-learning algorithm can be useful for predicting p*TERT* status in patients with WHO grade II glioma and may aid in glioma management.

## Introduction

Large-scale tumor genomics research has altered the perspective of tumor research by revealing a novel method for classification of central nervous system (CNS) tumors, especially for the most malignant primary brain tumor: gliomas. Currently, gliomas are primarily classified based on the molecular characteristics of tumor tissues according to the 2016 World Health Organization (WHO) classification of CNS tumors (1), with the status of these molecular biomarkers guiding the chemotherapy and radiation therapy strategies after surgical resection. Based on these new classification standards, glioblastomas and oligodendrogliomas often exhibit mutations in the telomerase reverse transcriptase promoter (p*TERT*) (1, 2). The function of *TERT* is to maintain telomere length, which shortens with each division of normal cells (3, 4). When p*TERT* is mutated, *TERT* is upregulated, resulting in maintenance of cellular growth (5). Mutations in p*TERT* can be detected in a variety of tumors. In high-grade glioma glioblastoma and low-grade glioma oligodendroglioma, mutations in p*TERT* can be detected with a high probability. According to the cIMPACT-NOW update, mutations in p*TERT* usually suggest a better prognosis in *IDH*-mutant diffuse gliomas. Conversely, mutations in p*TERT* in *IDH*-wild-type diffuse gliomas and glioblastomas suggest a poor prognosis (6). Thus, determining p*TERT* status can be helpful for predicting prognosis and optimizing clinical treatment targets.

Radiomics analysis has been widely adopted in the field of preoperative prediction in gliomas. The use of radiomics to analyze the WHO grades, molecular characteristics, and clinical outcomes of tumor tissue *via* preoperative magnetic resonance imaging (MRI) has produced good results (7–10). However, the predominant focus of many prior studies has been the prediction of the subtype combination of p*TERT* and *IDH*, which has demonstrated moderate performance (11, 12), rather than the status of p*TERT* alone. Other studies have exhibited superior performance at predicting p*TERT* status in patients, including those with higher-grade gliomas (WHO grade III or IV) (13, 14). In this regard, p*TERT* status in WHO grade II gliomas has rarely been predicted directly. In addition, the limited sample sizes used in previous prediction models pose several issues arising from overfitting when generalizing to other patient populations.

In the present study, we aimed to investigate the potential association between radiomics features and p*TERT* mutations by selecting valuable radiomics-based features. Based on extracted radiomics features from conventional MRI sequences used in most hospitals and clinical centers, we attempt to preoperatively predict the p*TERT* mutation status of WHO grade II gliomas by developing a machine-learning-based predictive model with limited overfitting and bias *via* a nested 10-fold cross-validation.

## Materials and Methods

### Patients

The clinical histories of 275 patients with pathologically confirmed primary WHO grade II gliomas were retrospectively collected from the CGGA database from June 2014 to June 2019. The following inclusion criteria were used: (a) adult (age ≥18 years); (b) histopathological diagnosis of primary grade II glioma; (c) no history of preoperative therapy or biopsy; and (d) available preoperative conventional MRI sequences, including T1-weighted images (T1WIs), T2-weighted images (T2WIs), and contrast-enhancement T1WIs (CE-T1WIs). Information on *IDH* and 1p/19q statuses was acquired from the CGGA database (http://www.cgga.org.cn/), and the details of the measurements and relationship among molecular biomarkers are shown in the **Supplementary Materials** and **Supplementary Table S1**, respectively.

### Ethics Statement

All clinical information was retrospectively collected from the institutional medical database, and the retrospective study was approved by the local institutional review board.

### Telomerase Reverse Transcriptase Promoter Mutation

Polymerase chain reaction (PCR) and Sanger sequencing were used to identify mutations in p*TERT* (15). The genomic mutational hotspots in the core promoter region of *TERT* were covered by sequences, including the nucleotide positions 1,295,228 [C228T] and 1,295,250 [C250T]. Nested PCR was performed for amplification based on the human genome reference sequence (grCh37 February 2009; http://genome.ucsc.edu/). To remove any unused primers, PCR products were purified using Illustra ExoProStar system (GE Healthcare, Buckinghamshire, UK) after amplification. The quality of PCR products was analyzed by electrophoresis on 2% agarose gels before sequencing. Then, PCR products were directly sequenced using a BigDye Terminator cycle sequencing kit on an ABI 3100 PRISM DNA sequencer (Applied Biosystems, Foster City, CA, USA).

### Magnetic Resonance Imaging Acquisition and Preprocessing

Regions of interest (ROIs) were drawn in slices presenting with tumors based on T2WI, in which the abnormal area could accurately represent the region implicated in low-grade gliomas (16–19). MRI was mainly performed using a Trio 3.0-T scanner (Siemens, Erlangen, Germany). T2WIs were obtained with the following imaging parameters: TR = 5,500 ms; TE = 120 ms; field of view = 240 × 240 mm^2^; flip angle = 150°; and voxel size = 0.65 × 0.65 × 5 mm^3^. T1WIs were obtained with the following parameters: TR = 450 ms; TE = 15 ms; field of view = 240 × 240 mm^2^; voxel size = 0.65 ×0.65 × 5 mm^3^. Patients were injected with gadopentetate dimeglumine intravenously (0.1 mM/kg), and CE-T1WIs were collected after contrast injection. To delineate tumor masks, two neurosurgeons (>5 years of experience, ZF and ZS) who were blinded to the patients’ clinical information used MRIcro (http://www.mccauslandcenter.sc.edu/mricro/) to draw the ROIs. Regions with hyperintense signals on T2WI were considered tumor areas. The T2WI and ROI for each patient were then registered to the high-resolution (1.0-mm isotropic) MNI (Montreal Neurological Institute) brain space using the SPM8 software (http://www.fil.ion.ucl.ac.uk/spm/software/spm8). A senior neuroradiologist (>20 years of experience, SL) made the final decision when the inter-neurosurgeon’s discrepancies of tumor masks exceeded 5% (DICE coefficient).

### Quantitative Radiological Feature Extraction

**Figure 1:**
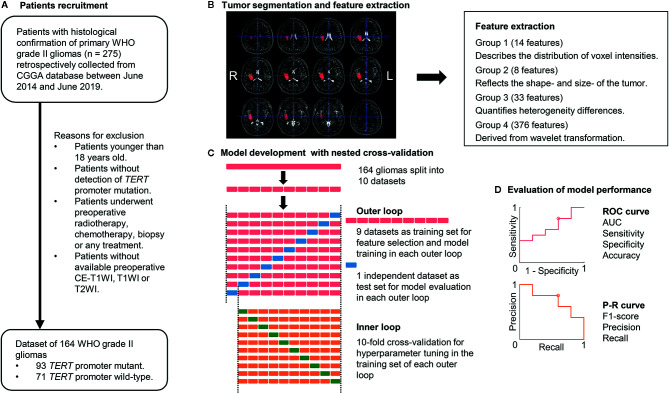
Workflow of patient recruitment, image processing, and machine-learning. **(A)** Patient recruitment process. **(B)** Image processing. Tumor segmentation was performed with T2-weighted images (T2WIs). Radiomics features were extracted from T1-weighted images (T1WIs), T2WIs, and contrast-enhanced T1WI using region-of-interest masks. **(C)** Feature selection and machine-learning were computed in a nested 10-fold cross-validation scheme, which comprised an inner and outer loop. The inner loop included hyperparameter tuning and feature selection. The outer loop was performed for the evaluation of model performance. **(D)** ROC analysis and P-R analysis were used for model performance evaluation. *AUC*, area under the curve; *CGGA*, Chinese Glioma Genome Atlas database*; TERT*, telomerase reverse transcriptase; *P-R analysis*, precision and recall analysis; *ROC*, receiver operating characteristic.

To avoid heterogeneity bias, various MRI signal intensity values were transformed into standardized intensity ranges *via* z-score transformation. Radiomics features were then extracted from tumor masks based on the different types of MRI sequences using an automated approach (details are provided in the **Supplementary Material**) (20). For each sequence, 431 radiomics features were extracted and classified into four types (**Figure 1**): (1) first-order statistics features (n = 14), which quantitatively delineate the distribution of voxel intensities with the MRI scan through commonly used and basic metrics; (2) shape- and size-based features (n = 8), which used three dimensional features to reflect the shape and size of the ROI; (3) textural features (n = 33), which are calculated from gray-level run-length and gray level co-occurrence texture metrics and reflect the intra-tumoral heterogeneity differences; and (4) wavelet features (n = 376), which were transferred from intensity and texture features.

### Feature Selection and Model Development

We developed a commonly used machine-learning algorithm, the linear support vector machine (linear SVM), to build predictive models. A linear SVM, which specified the use of a linear kernel, aimed to identify the best hyperplane that maximizes the margin between the data points of two classes (21–25). The *fitcsvm* function in MATLAB was used to build the linear SVM model. To optimize the predictive models, we varied the box constraint and kernel scale parameters in a 10-fold cross-validation (CV). In the CV, predictive models with minimal loss were considered as the optimal model.

The linear SVM was evaluated with a nested k-fold CV approach. Nested CV is widely employed in the machine-learning analysis of neuro-imaging (12, 26–29). Compared to simple CV, nested CV can reduce overfitting and limit optimistic biases, especially in relatively small samples (30, 31). These methods can make full use of all the information in the dataset and prevent circular analysis. After the dataset is split into 10 non-overlapping subsets, one selected subset (test dataset) is used to estimate the performance of a model that is trained by the remaining nine subsets (training dataset), which used another 10-fold CV for hyperparameter tuning (inner loop). These processes are repeated 10 times (outer loop), each time selecting an independent subset as the test dataset for model evaluation. We performed a 10-fold CV in the outer loops and computed the model performance, which was evaluated by ROC and PR analyses, using posterior probabilities.

Feature selection using elastic net (E-net) was conducted in the training component with nine datasets of each outer loop. The E-net penalty was regarded as a weighted sum of the least absolute shrinkage and selection operator penalty (LASSO) and ridge penalty (32, 33). *λ* and *α*, ranging from 0 to 1 in steps of 0.1, were selected using 10-fold CV *via* minimum or minimum plus one standard error criteria in the E-net model. We then selected the valuable features with non-zero coefficient resulting from the optimal *λ* and *α* for further analysis. After feature selection, a linear SVM was trained using the training dataset with an inner 10-fold CV loop for hyperparameter tuning. Grid searches were used for all of the hyperparameter tuning processes. Thus, 10 different linear SVM models were built with specific sets of features and hyperparameters.

### Statistical Analyses

The entire nested 10-fold CV process was computed in MATLAB 2019b (MathWorks, Natick, MA, USA). Receiver operating characteristic (ROC) and precision-recall analyses were conducted to determine the performance of models in the prediction of p*TERT* status. The optimal threshold was identified when the sum of sensitivity and specificity was maximal. The 95% confidence interval (CI) of performance was evaluated using bootstrapping. We report the correlation coefficients and corresponding *p* values of the point-biserial-correlation between the true labels and posterior probabilities of *TERT* status, which were transformed from the decision values of SVM (34). The linear SVM model decision values of patients with wild-type or mutant p*TERT* were compared using unpaired t-test. Data are presented as means ± standard deviations. Differences were considered statistically significant at a P-value (*p*) <0.05.

## Results

### Clinical Characteristics

Overall, 275 patients with pathological confirmed primary WHO grade II gliomas were retrospectively collected from the CGGA database. We excluded 26 patients younger than 18 years of age; 11 patients without results of *TERT* promoter mutation; eight patient received radiotherapy; chemotherapy, biopsy, or any treatment before preoperative MRI examinations; and 66 patients without available preoperative MRIs. As a result, we retrospectively enrolled 164 patients with primary WHO grade II gliomas (89 men and 75 women; age range, 20–80 years; **Table 1**). The proportion of patients with a mutation in p*TERT* was 56.7% (93/164). The proportion of patients with a mutation in *IDH* and a 1p/19q codeletion were 86% (141/164) and 48.2% (79/164), respectively. The mean ( ± standard deviation) age and tumor volume were 41.6 ± 10.4 years and 61.4 ± 55.3 cm^3^, respectively.

**Table 1:** Baseline demographics and clinical characteristics of patients.

Variable	Value
Number of Patients	164
Sex	
Male	89
Female	75
Age (years)*	41.6 ± 10.4
*IDH*	
Wild-type	23
Mutant	141
1p/19q	
Codeletion	79
Non-codeletion	85
p*TERT*	
Wild-type	71
Mutant	93
Tumor volume (cm^3^)	61.4 ± 55.3

*Data are presented as means ± standard deviations.

IDH, isocitrate dehydrogenase; NOS, not otherwise specified; pTERT, telomerase reverse transcriptase promoter.

### Radiomics Feature Selection

We extracted 431 features from each sequence and a total of 1,293 radiomics features from all conventional sequences for each patient. The radiomics features selected by E-net in each outer loop ranged from 12 to 234. Features that were selected in at least nine of the 10 loops were considered to be the most valuable (**Table 2**). The 12 valuable radiomics features that were retained were textual features (Group 3) and their wavelet-transformed features (Group 4), such as CE-T1WI_Cluster Tendency, T1WI_Contrast, T1WI_Long Run Low Gray Level Emphasis_1, T1WI_Low Gray Level Run Emphasis, T2WI_Long Run High Gray Level Emphasis_1, *etc.* The z-score-transformed value of each important radiomics feature and p*TERT* status were compared, revealing that all valuable radiomics features in patients with mutations in p*TERT* were significantly different from patients with wild-type p*TERT* (*p* < 0.05).

**Table 2:** Selected valuable features.

Feature name	Selected times	p*
CE-T1WI_Cluster Tendency (Group 3)	10	0.0025
T1WI_Contrast (Group 3)	10	<0.0001
T1WI_Long Run Low Gray Level Emphasis_1 (Group 4)	10	<0.0001
T1WI_Low Gray Level Run Emphasis (Group 3)	10	0.0066
T2WI_Long Run High Gray Level Emphasis_1 (Group 4)	10	<0.0001
CE-T1WI_Homogeneity 2_4 (Group 4)	9	0.0055
CE-T1WI _Sum Entropy_1 (Group 4)	9	<0.0001
CE-T1WI _Sum Variance_2 (Group 4)	9	0.0056
CE-T1WI _Variance_2 (Group 4)	9	<0.0001
T1WI_Cluster Prominence (Group 3)	9	<0.0001
T1WI_ Inverse Difference Moment Normalized (Group 3)	9	0.0006
T2WI_Homogeneity 2 (Group 3)	9	0.0007

*P-value of comparison between TERT promoter mutant and wild-type using unpaired t-test.

### Model Performance

**Figure 2:**
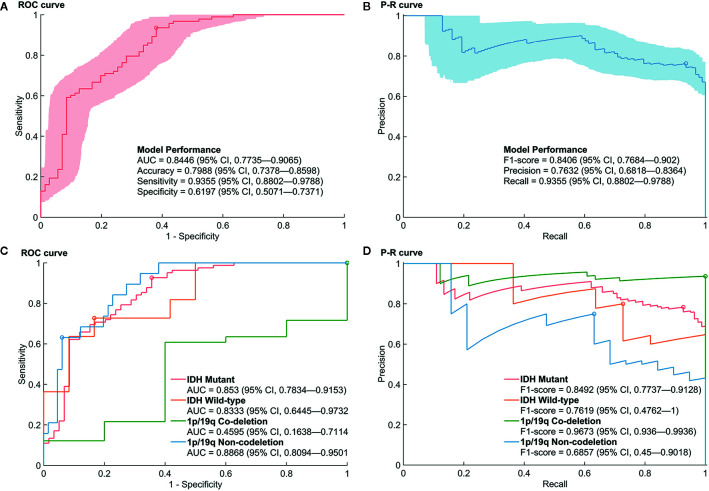
Performance of the prediction model for mutations in the promoter region of *TERT* (p*TERT*) in WHO grade II gliomas. **(A, B)** The receiver operating characteristic (ROC) curve and P–R curve in the prediction of mutations in p*TERT* in WHO grade II gliomas. **(C, D)** The ROC curve and P–R curve in the prediction of mutations in p*TERT* in the subgroups of molecular biomarkers in WHO grade II gliomas.

**Figure 3:**
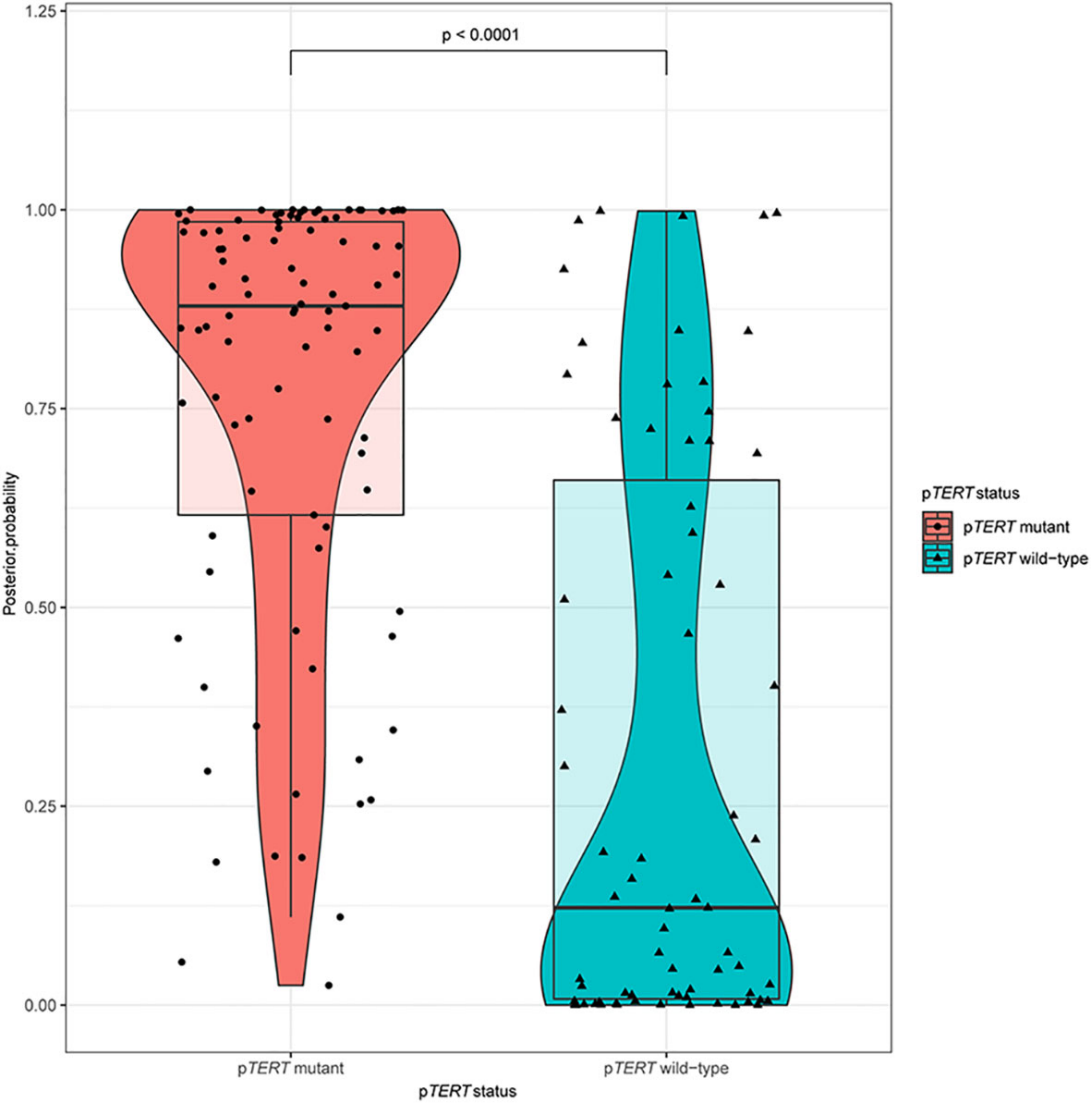
A violin plot comparing the differences in posterior probabilities between mutants and wild-types promoter regions of *TERT*.

Ten predictive models were built in this study. Model parameters are shown in **Table 3**, and the performance of each predictive model in each loop are summarized in **Supplementary Table S2**. The box constraints and kernel scale ranged from 10 to 1,000 and 0.46 to 215.4, respectively. The ROC analysis revealed an AUC value of 0.8446 (95% CI, 0.7735–0.9065), with optimal summed values of sensitivity of 0.9355 (95% CI, 0.8802–0.9788) and specificity of 0.6197 (95% CI, 0.5071–0.7371) (**Figure 2**). The overall accuracy was 0.7988 (95% CI, 0.7378–0.8598). The P-R analysis displayed an F1-score value of 0.8406 (95% CI, 0.7684–0.902) with an optimal precision of 0.7632 (95% CI, 0.6818–0.8364) and optimal recall of 0.9355 (95% CI, 0.8802–0.9788). A total of 34 patients were misclassified. There were 27 (79.4%) patients with wild-type p*TERT*, 28 (82.4%) patients with wild-type *IDH*, and 26 (76.5%) patients with 1p/19q non-codeletion. To evaluate the association between posterior probability and true labels, we computed point-biserial-correlations, revealing r and *p* values of 0.59 and <0.0001, respectively. Further, we compared the posterior probability between wild-type and mutant p*TERT*, revealing a *p-*value <0.0001, which indicated that our model can be used to predict the p*TERT* status of WHO grade II gliomas (**Figure 3**).

**Table 3:** Optimal model parameters in each outer loop.

Loops	Box constraint	Kernel scale
Loop 1	1,000	215.4
Loop 2	215.4	46.4
Loop 3	46.4	46.4
Loop 4	1000	215.4
Loop 5	215.4	10
Loop 6	10	10
Loop 7	1,000	46.4
Loop 8	46.4	46.4
Loop 9	1,000	215.4
Loop 10	10	0.46

Furthermore, the performances of the prediction model in the subgroup of *IDH* and 1p/19q were evaluated. Although the AUCs of the ROC analysis reached a value of 0.853 (95% CI, 0.7834–0.9153), 0.8333 (95% CI, 0.6445–0.9732), and 0.8868 (95% CI, 0.8094–0.9501) for mutant *IDH*, wild-type *IDH*, and 1p/19q non-deletion groups, respectively, the AUC for the 1p/19q codeletion showed a rather low value of 0.4595 (95% CI, 0.1638–0.7114). Regarding the high rate of p*TERT* mutations in the 1p/19q codeletion (74/79), the P–R Curve, which is suitable for describing imbalances in binary data, showed a more reliable result to evaluate the model performance. The precision, recall, and F1-score in 1p/19q codeletion group were 0.9367 (95% CI, 0.8797–0.9873), 1 (95% CI, 1–1), and 0.9673 (95% CI, 0.936–0.9936), respectively. In addition, the accuracies were 0.8085 (95% CI, 0.7447–0.8652), 0.7826 (95% CI, 0.6087–0.913), 0.9367 (95% CI, 0.8734–0.9873), and 0.8706 (95% CI, 0.8–0.9412) in mutant *IDH*, wild-type *IDH*, 1p/19q codeletion, and 1p/19q non-deletion groups, respectively. The detailed prediction model performances in molecular subgroups are shown in **Supplementary Table S3**.

## Discussion

The clinical characteristics of patients with mutations in p*TERT* were associated with poor prognosis with glioblastomas and a good prognosis with oligodendroglioma (6, 35, 36). Based on the presence of p*TERT* mutations, *IDH1/2* mutations, and 1p/19q codeletion status, gliomas were divided into five subtypes with different overall survival (37). Since patients with lower-grade gliomas (LGGs) who carry mutations in p*TERT* always have a better survival, the determination of p*TERT* status by a non-invasive MRI scan may help patients make better decisions regarding their treatment plan. The development of an efficient method to accurately and preoperatively identify the p*TERT* status of the tumor before surgery is a critical unmet need. In this regard, radiomics offers a promising approach. To predict p*TERT* mutation status preoperatively, we built a preoperative model based on radiomics analysis that exhibited good performance and robustness.

Based on artificial intelligence, radiomics showed its potential to connect radiological images and tumor metadata (38). Radiological images contain tumor features, such as shape, volume, density, structure, and other characteristics, which are associated with tumor genomics (39). In this study, data from 164 patients with WHO grade II gliomas, whose p*TERT* status and preoperative MRIs were available, were included into the dataset. We used E-net to reduce dimensionality, identified the main features of the data, and attempted to eliminate the overfitting phenomenon of the model caused by excessive features (40). In total, 12 important radiomics features were selected more than nine times by E-net in the loops. These valuable radiomics features were predominantly textual information that could not be fully identified by the human eye in imaging and reflected the internal tissue characteristics of tumor imaging, such as internal density, morphological cell proliferation state, and infiltration degree (41–44). Among the five top radiomics features selected by the loops (10 times), three features were selected from T1WIs, and one feature was selected from CE-T1WIs and T2WIs, respectively. These results indicated that T1WIs provide the most valuable information for predictions given that WHO grade II gliomas are rarely contrast-enhanced.

By analyzing the textures extracted from patients’ radiological images, substantial progress has been made with regard to WHO grade and genotype prediction of gliomas (7, 9, 16, 45–48). On one hand, since the classification of p*TERT* status has predominantly been associated with *IDH* and 1p/19q alterations, previous studies have aimed at combining subtypes of mutations in p*TERT* and *IDH* mutations for predictions. However, these attempts did not achieve a satisfactory result. Based on the radiomics analysis of conventional MRI, a LASSO regression model was used for predicting molecular subtypes of LGGs including mutant *IDH1/2*, mutant *IDH1/2* with *p*TERT mutations, and wild-type *IDH* (11). The accuracies of the prediction model reached 0.74 in the training set and 0.56 in the validation set. Another study showed lower performance based on the combination of patient age, radiomics features, and convolutional neural network features; a linear SVM model was used for predicting three subtypes of LGGs, and the accuracy reached 0.63 ± 0.08 (12). On the other hand, some studies presenting radiomics analysis focus on p*TERT* mutations only. A previous study compared three machine-learning methods in predicting p*TERT* mutations in LGGs, including random forest, SVM, and adaboost methods (13). The results showed that the random forest method had the best performance after feature selection using LASSO, and the AUC value reached 0.827 (95% CI, 0.667–0.988) in the validation group. An extreme gradient boosting model with recursive feature selection showed similarity AUC of 0.82 ± 0.04 (29), which was similar to our prediction models. In addition, based on the convolutional neural network features described above, the linear SVM model reached an accuracy of 0.84 ± 0.09 (12). Further, the prediction of mutations in p*TERT* in the subgroup of *IDH* also reached stable performances, where the random forest model achieved an AUC of 0.824 (95% CI, 0.639–1) and 0.750 (95% CI, 0.260–1) in the mutant *IDH* and wild-type *IDH* groups, respectively (13).

Although the above radiomics-based analysis achieved good performance in the predication of mutations in p*TERT*, previous studies have focused on LGGs, which are composed of WHO grades II and III gliomas, with limited sample sizes (11–13, 29). However, gliomas in WHO grade II and HGG showed differences in biological and radiomics features (49). Thus, the present study focuses on WHO grade II gliomas, which decreased the sample size but improved the consistency and practicality of the results. As a result, we enrolled 164 patients with WHO grade II gliomas and used nested CV to fully utilize the information of the enrolled patients. In addition, the performance of the prediction was also evaluated in the subgroups with *IDH* and 1p/19q alterations, which reached high and stable accuracies. However, because of the highly skewed dataset of the 1p/19q codeletion group (74 p*TERT* mutant and five wild-type samples), the ROC curve was limited, and the P–R curve gave a more informative picture of performance (50), which showed a high F1-score of 0.9673 (95% CI, 0.936–0.9936) and a high accuracy of 0.9367 (95% CI, 0.8734–0.9873).

There are some limitations of this study. First, as all patients enrolled were from a single hospital, multi-center data verification is lacking. In subsequent experiments, we will include other clinical centers or glioma imaging datasets, such as TCIA, to eliminate potential systematic errors caused by using different equipment to collect image information. Second, ROI labeling in this study relied on manual labeling by imaging scientists, which inevitably resulted in differences in ROI interpretation and affected subsequent analysis and processing. To overcome this limitation, artificial intelligence labeling should be introduced in future research to automatically label ROIs and improve the efficiency and consistency of the prediction system.

In conclusion, our results demonstrate the clinical utility of radiomics analysis for predicting p*TERT* mutation status preoperatively. Through nested CV, we developed an efficient machine-learning-based model with robust performance. Given that p*TERT* mutation status plays an important role in glioma patients’ outcomes, our predictive model will facilitate the optimization of clinical management strategies for patients with gliomas.

## Data Availability Statement

The raw data supporting the conclusions of this article will be made available by the authors, without undue reservation.

## Ethics Statement

The studies involving human participants were reviewed and approved by the ethics committee of Beijing Tiantan Hospital (Beijing Tiantan Hospital, Capital Medical University, Beijing 100070, China). The patients/participants provided their written informed consent to participate in this study.

## Author Contributions

YW, TJ, and LW conceptualized and designed the study. SF, XL, YCL, YKL, CZ, QZ, TL, and HZ acquired the data. ZF, SF, ZS, YKL, SL, and YW analyzed and interpreted the data. SF and ZF drafted the manuscript. YW and LW critically revised the manuscript. All authors contributed to the article and approved the submitted version.

## Funding

This study was supported by grants from the Beijing Nova Program (No. Z181100006218064), Beijing Municipal Natural Science Foundation (No. 7202021), and Capital’s Funds for Health Improvement and Research (CFH 2018-2-1072).

## Conflict of Interest

The authors declare that the research was conducted in the absence of any commercial or financial relationships that could be construed as a potential conflict of interest.
