# Supplementary material for: Radiomics Features Predict Telomerase Reverse Transcriptase Promoter Mutations in World Health Organization Grade II Gliomas via a Machine-Learning Approach
Source: Front Oncol. 2021 Feb 11;10:606741. doi: 10.3389/fonc.2020.606741 (PMC7905226; doi:10.3389/fonc.2020.606741)
Supplement: Supplementary file 2 [file Table_1.docx]

**Supplementary Table S1**. The characteristics of average age,gender, IDH mutation status, and 1p/19q co-deletion status of patients in this study.

|  | **p*TERT*-mut (93)** | | **p*TERT*-wt (71)** | |
| --- | --- | --- | --- | --- |
| Average age (year) | 42.68 | | 40.3 | |
| Gender | 47 (M) | 46 (F) | 42 (M) | 29 (F) |
| IDH status | 82 (mut) | 11 (wt) | 59 (mut) | 12 (wt) |
| 1p/19q co-deletion | 74 (co-del) | 19 (non-co) | 5 (co-del) | 66 (non-co) |

Abbreviations: *IDH*, isocitrate dehydrogenase; p*TERT*, telomerase reverse transcriptase promoter; mut, mutant; wt, wild-type; M, male; F, female; co-del, co-deletion; non-co, non-codeletion.
