# Supplementary material for: Radiomics Features Predict Telomerase Reverse Transcriptase Promoter Mutations in World Health Organization Grade II Gliomas via a Machine-Learning Approach
Source: Front Oncol. 2021 Feb 11;10:606741. doi: 10.3389/fonc.2020.606741 (PMC7905226; doi:10.3389/fonc.2020.606741)
Supplement: Supplementary file 3 [file Table_2.docx]

**Supplementary Table S2.** Performances of p*TERT* mutation prediction models in each loop

| **Loops** | **AUC** | **Accuracy** | **Specificity** | **Sensitivity/Recall** | **Precision** | **F1-score** |
| --- | --- | --- | --- | --- | --- | --- |
| 1 | 0.78 (0.49—0.98) | 0.75 (0.5—0.94) | 0.71 (1—0.33) | 0.78 (0.5—1) | 0.78 (0.44—1) | 0.78 (0.47—1) |
| 2 | 0.92 (0.72—1) | 0.88 (0.71—1) | 0.88 (1—0.6) | 0.89 (0.67—1) | 0.89 (0.63—1) | 0.89 (0.65—1) |
| 3 | 0.74 (0.46—0.97) | 0.76 (0.53—0.94) | 0.43 (0.83—0) | 1 (1—1) | 0.71 (0.48—0.93) | 0.83 (0.65—0.97) |
| 4 | 0.87 (0.64—1) | 0.82 (0.65—1) | 0.86 (1—0.5) | 0.8 (0.55—1) | 0.89 (0.67—1) | 0.84 (0.6—1) |
| 5 | 0.76 (0.46—1) | 0.82 (0.65—1) | 0.57 (1—0.17) | 1 (1—1) | 0.77 (0.54—1) | 0.87 (0.7—1) |
| 6 | 0.62 (0.29—0.9) | 0.75 (0.5—0.94) | 1 (1—1) | 0.56 (0.22—0.88) | 1 (1—1) | 0.71 (0.36—0.93) |
| 7 | 0.98 (0.9—1) | 0.94 (0.78—1) | 1 (1—1) | 0.89 (0.63—1) | 1 (1—1) | 0.94 (0.77—1) |
| 8 | 1 (1—1) | 1 (1—1) | 1 (1—1) | 1 (1—1) | 1 (1—1) | 1 (1—1) |
| 9 | 0.92 (0.72—1) | 0.88 (0.69—1) | 0.86 (1—0.53) | 0.89 (0.64—1) | 0.89 (0.64—1) | 0.89 (0.64—1) |
| 10 | 0.97 (0.87—1) | 0.94 (0.81—1) | 0.86 (1—0.5) | 1 (1—1) | 0.9 (0.69—1) | 0.95 (0.82—1) |
