# Supplementary material for: Radiomics Features Predict Telomerase Reverse Transcriptase Promoter Mutations in World Health Organization Grade II Gliomas via a Machine-Learning Approach
Source: Front Oncol. 2021 Feb 11;10:606741. doi: 10.3389/fonc.2020.606741 (PMC7905226; doi:10.3389/fonc.2020.606741)
Supplement: Supplementary file 4 [file Table_3.docx]

**Supplementary Table S3.** Performances of p*TERT* mutation prediction models in subgroups

| **Subgroups** | **AUC** | **Accuracy** | **Specificity** | **Sensitivity/Recall** | **Precision** | **F1-score** |
| --- | --- | --- | --- | --- | --- | --- |
| IDH mutant | 0.85 (0.78—0.91) | 0.81 (0.74—0.87) | 0.64 (0.53—0.76) | 0.93 (0.87—0.98) | 0.78 (0.69—0.86) | 0.85 (0.77—0.92) |
| IDH wild-type | 0.83 (0.63—0.98) | 0.78 (0.61—0.91) | 0.83 (0.57—1) | 0.73 (0.42—1) | 0.8 (0.5—1) | 0.76 (0.47—1) |
| 1p/19q co-deletion | 0.46 (0.21—0.71) | 0.94 (0.87—0.99) | 0 (0—0) | 1 (1—1) | 0.94 (0.89—0.99) | 0.97 (0.94—0.99) |
| 1p/19q non-codeletion | 0.89 (0.81—0.95) | 0.87 (0.8—0.94) | 0.94 (0.88—0.99) | 0.63 (0.4—0.86) | 0.75 (0.5—0.95) | 0.69 (0.45—0.89) |
